# Genetics, pathogenicity and transmissibility of novel reassortant H5N6 highly pathogenic avian influenza viruses first isolated from migratory birds in western China

**DOI:** 10.1038/s41426-017-0001-1

**Published:** 2018-01-24

**Authors:** Shaoxia Lu, Zongzheng Zhao, Jiajie Zhang, Weidong Wang, Xin He, Mengqi Yu, Chunmao Zhang, Xiang Li, Zhendong Guo, Xiaoyu Yang, Lina Liu, Min Zhi, Tian Fu, Xinru Lv, Wenge Ma, Mengying Liao, Hongliang Chai, Linna Liu, Jun Qian, Jianzhang Ma

**Affiliations:** 1 College of Wildlife Resources, Northeast Forestry University, Harbin, 150040 Heilongjiang China; 2 Military Veterinary Research Institute of Academy of Military Medical Sciences, Changchun, 130062 Jilin China; 3 Monitoring Center for Terrestrial Wildlife Epidemic Diseases, Ningxia, Yinchuan, 750001, Ningxia Hui Autonomous Region China

Novel H5N6 viruses were first documented in Laos in 2013 and subsequently reported in Vietnam and China^1^, but these viruses did not initially attract a great deal of attention. Instead, there was a greater focus on known H5N6 subtype AIVs until a fatal human infection with a novel H5N6 virus was confirmed in Sichuan Province, China^2^. As of May 2017, 17 human infections with these novel H5N6 highly pathogenic avian influenza viruses (HPAIVs), including 12 deaths, have been identified^3^.

H5N6 was identified as one of the dominant AIV subtypes among poultry in southern China^4^. However, as of June 2017, few cases involving wild birds infected with H5N6 AIVs have been reported in China, and these cases have been reported only in central (Hubei) and eastern China (Jilin, Guangdong)^2,4,5^. We first reported novel H5N6 HPAIVs infections in eight species of migratory birds in western China.

In November 2015, oropharyngeal and cloacal swabs from 80 wild waterfowl were collected during active surveillance at Changshantou reservoir, Ningxia, western China (Fig. 1a). Viruses were isolated in 10-day-old specific pathogen-free chicken embryos, and 17 samples were positive for H5N6 avian influenza virus according to RT-PCR detection; thus, the separation rate was 21.25% (17/80). All sequences determined in this study were confirmed by Sanger sequencing and have been submitted to GenBank under accession numbers MF399537–MF399672. We performed further experiments, including a receptor-binding specificity assay, a mouse pathogenicity test, and a guinea pig transmission test, to evaluate the pathogenicity and transmissibility of the isolated viruses (Supplementary Materials).

**Fig. 1:**
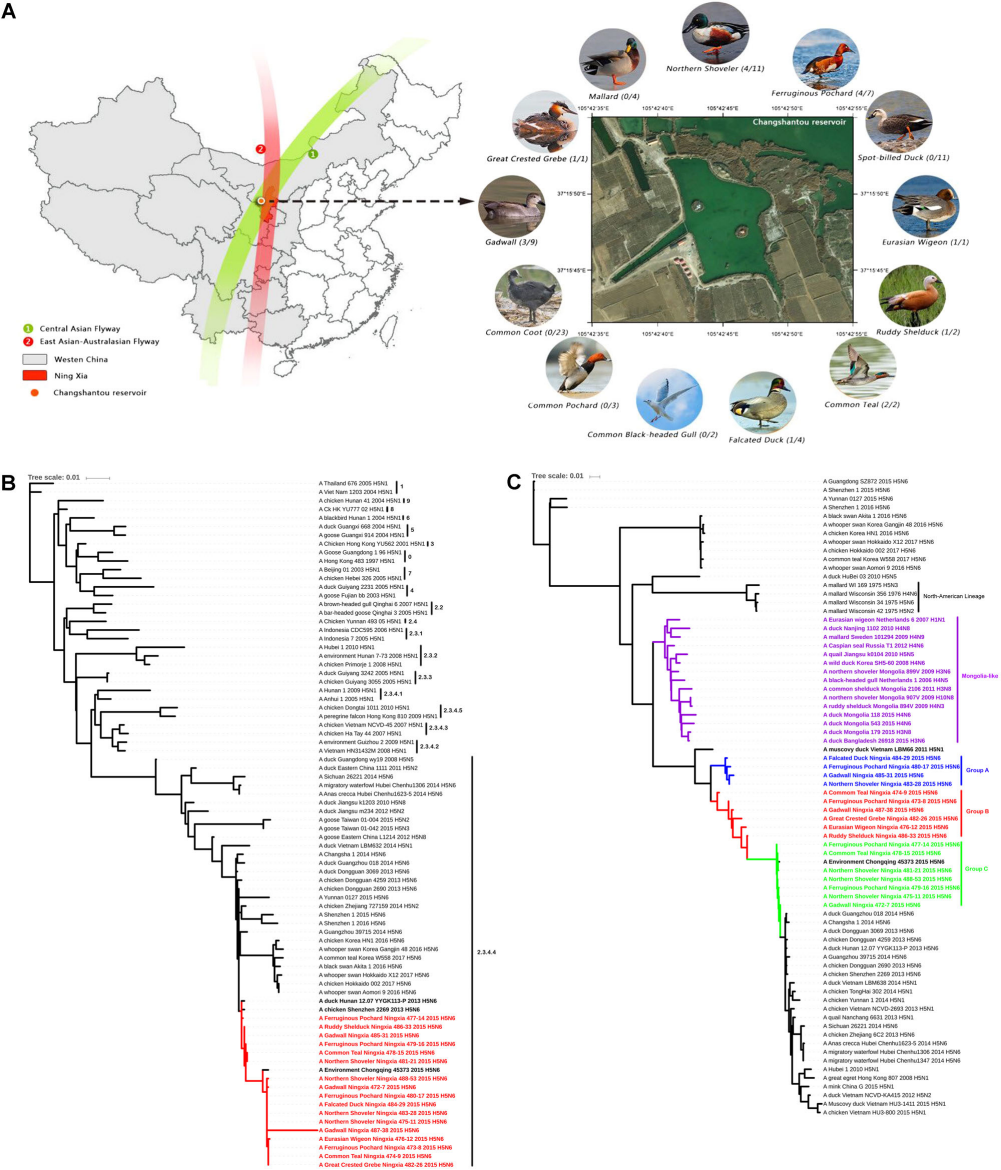
The sampling location and phylogenetic analysis of *HA* and *PB1* genes. **a** The sampling location in Ningxia, western China. The ratio of the number of isolated viruses to the number of samples is indicated in parentheses. **b** Phylogenetic tree of HA showing relationships of emergent influenza A(H5N6) viruses with clade 2.3.4.4 H5 avian influenza viruses and **c** phylogenetic tree of PB1 showing that 17 PB1 genes cluster into three groups named Group A (red), Group B (green), and Group C (blue), evolving from Mongolia-like LPAIVs

The HA proteins of all 17 H5N6 AIVs contain the HPAIV amino acid sequence RERRRKR/GLF at the site of cleavage into HA1 and HA2^6^. In these viruses’ HA proteins, although the amino acid substitutions Q222L and G224S (H5 numbering) were not found, the substitutions D94N, S123P, and S133A (H5 numbering), which are associated with increased binding to α-2, 6-linked sialic acid were identified^7^. Moreover, an 11-aa deletion at residues 59–69 of the neuraminidase (NA) protein was identified in all isolated viruses; this deletion could enhance virulence in mammals^8^. Mutations associated with increased virulence in mice were observed in matrix 1 protein (N30D)^9^, polymerase basic 2 protein (L89V), and nonstructural protein 1 (P42S, D87E, L98F, I101M, and the 80–84 deletion) in all isolated viruses (Supplementary Table S1)^10,11^.

Phylogenetic analysis was performed using RAxML with 1000 bootstrap replicates, which revealed that all 17 isolated virus genomes belong to the Eurasian lineage (Fig. 1b–c and Supplementary Fig. S1A–F), and these viruses’ hemagglutinin (HA) genes clustered into clade 2.3.4.4 (Fig. 1b). Seven genes of the 17 isolated viruses, except the polymerase basic 1 (*PB1*) gene, originate from A/duck/Hunan/12.07 YYGK113-P/2013(H5N6) (HN113-P) and share the highest nucleotide identity with A/Environment/Chongqing/45373/2015(H5N6) (CQ45373), with identities ranging from 98.9 to 99.6% (for the *HA* gene, with the exception of the *HA* gene of NX488-38) to 99.8–100% (for the *NS* gene), indicating the same genotype. In particular, the *HA* gene of A/Gadwall/Ningxia/487-38/2015(H5N6) (NX487-38) shares less than 97.6% nucleotide identity with all known strains. All isolated viruses also share high homology with A/Changsha/1/2014(H5N6) (CS1), with 98.2–99.2% nucleotide identity for the *HA* gene and 98.4–99.7% nucleotide identity for other genes, with the exception of the *PB1* gene and *HA* gene of NX487-38.

The nucleotide homologies for the *PB1* genes of the 17 isolated viruses were 92.8–99.9%; therefore, these *PB1* genes exhibit diversity. A phylogenetic tree of *PB1* genes showed that all 17 isolated viruses clustered into three groups (Groups A, B, and C) (Fig. 1c). Group A shares less than 93% nucleotide homology with Group C, and Group B is a transition group between Group A and Group C. Group B and Group C share the highest nucleotide homologies with CQ45373. A phylogenetic tree showed that the *PB1* gene may originate from unidentified Mongolia-like low pathogenic avian influenza viruses (LPAIVs), evolving from A/duck/Mongolia/179/2015 (H3N8) and A/duck/Bangladesh/26918/2015 (H3N6), but the detailed evolutionary history of *PB1* genes remains unclear.

The isolates are closely related to human virus CS1, and sequence analysis showed that they possess the same key amino acids associated with mammalian infectivity and pathogenicity. Therefore, we selected NX488-53 (because all isolated viruses have the same key amino mutations, as shown in Supplementary Table S1) as a representative strain and performed further experiments. The receptor-binding specificity was determined by hemagglutinin assays, revealing that the NX488-53 virus preferentially binds α-2, 3-linked avian sialic acid receptors (Supplementary Fig. S2). We further evaluated the pathogenicity of NX488-53 in a mouse model (Supplementary Materials). The body weights of the virus-inoculated BALB/c mice first decreased and then increased gradually. At 14 dpi, the body weights of the infected mice were comparable to those of the controls (Supplementary Fig. S3A). The pathogenicity of the NX488-53 virus in the inoculated BALB/c mice was low and thus resulted in non-lethal infections (Supplementary Fig. S3B). The virus replicated well in the lungs of infected mice at 1, 3, 5, and 7 dpi, with titers ranging from 101.8 to 103.2 EID_50_ (Supplementary Fig. S3C). The virus was also detected in the kidney, heart, and liver at 5 dpi, but no virus was detected in the brain or spleen throughout the course of infection (Supplementary Fig. S3C). Histological analysis showed that the NX488-53 virus was capable of systemic infection and induced slight pathological changes (Supplementary Fig. S4). These results indicate that mice could be infected by this virus without prior adaption, but the virus showed low pathogenicity in BALB/c mice. A guinea pig transmission test showed that the virus was detected in only nasal washes of the group inoculated intranasally (Supplementary Fig. S5), indicating that it had not yet acquired transmissibility among guinea pigs via direct contact or aerosol.

We report the first case of novel H5N6 HPAIV infection in migratory birds in western China. Phylogenetic and molecular analyses indicated that all novel H5N6 HPAIVs have been generated by the reassortants of Mongolia-like LPAIVs and HN113-P, which donated their *PB1* gene and another seven genes, respectively. These viruses’ *HA* genes cluster into clade 2.3.4.4. The *PB1* genes from the isolated viruses exhibit diversity.

Although NX488-53 showed low pathogenicity in BALB/c mice, mice could be infected by this virus without prior adaption. These results indicate that the isolated viruses may be able to infect other mammals, including humans. In addition, an examination of the transmissibility of the NX488-53 virus in guinea pigs showed that it has not acquired the ability to be transmitted among guinea pigs via direct contact or aerosol, indicating that these viruses may not yet have the capacity for mammal-to-mammal transmission, which is one possible reason for the sporadic H5N6 infections in humans, as opposed to large-scale outbreaks.

Wild aquatic birds have been suspected to play a key role in the dissemination of H5 HPAIVs to various regions during their migration, such as clade 2.2 H5N1 HPAIV in 2005, clade 2.3.2.1 H5N1 HPAIV in 2009, and clade 2.3.4.4 H5N8 HPAIV in 2014^12,13^. Ningxia, which is located in western China, is where the East Asian/Australasian and Central Asian flyways overlap and therefore represents an important ecological niche for migratory birds (Fig. 1a). In this study, *PB1* genes were found to involve from unidentified Mongolia-like LPAIVs; thus, we speculate that the isolated viruses may have been generated by the reassortment of circulating H5N6 viruses in China with Mongolia-like LPAIVs, suggesting possible close contact during migration of birds carrying LPAIVs from Mongolia to wintering sites. Therefore, in addition to intensification of the surveillance of AIVs in migratory birds in western China, international surveillance and information sharing should be strengthened.

## Electronic supplementary material

Supplementary Figure S1

Supplementary Figure S2

Supplementary Figure S3

Supplementary Figure S4

Supplementary Figure S5

Supplementary materials

Supplementary Table S1
