# Correction

**DOI:** 10.1080/22221751.2019.1583924

**Published:** 2019-03-08

**Article title:** Genetics, pathogenicity and transmissibility of novel reassortant H5N6 highly pathogenic avian influenza viruses first isolated from migratory birds in western China

**Authors:** Lu, S., Zhao, Z., Zhang, J., Wang, W., He, X., Yu, M., Zhang, C., Li, X., Guo, Z., Yang, X., Liu, L., Zhi, M., Fu, T., Lv, X., Ma, W., Liao, M., Chai, H., Liu, L., Qian, J., & Ma, J.

**Journal:***Emerging Microbes & Infections*

**Bibliometrics:** Volume 7 (2018)

**DOI:**https://doi.org/10.1038/s41426-017-0001-1

When this paper was first published online, an error was found in the following sentence on page 3:

The receptorbinding specificity was determined by hemagglutinin assays, revealing that the NX488-53 virus preferentially binds α-2, 3-linked avian sialic acid receptors (Supplementary Fig. S2)

The correct sentence should read:

The receptorbinding specificity was determined by hemagglutinin assays, revealing that the NX488-53 virus preferentially binds α-2, 6-linked avian sialic acid receptors (Supplementary Fig. S2)

This has now been corrected.

