# Supplementary material for: Genetics, pathogenicity and transmissibility of novel reassortant H5N6 highly pathogenic avian influenza viruses first isolated from migratory birds in western China
Source: Emerg Microbes Infect. 2018 Jan 24;7:6. doi: 10.1038/s41426-017-0001-1 (PMC5837145; doi:10.1038/s41426-017-0001-1)
Supplement: Supplementary file 1 — Supplementary Figure S1 [file 41426_2017_1_MOESM1_ESM.doc]

**Supplementary Figure S1**.Phylogenetic analyses of influenza A(H5N6) virus detected in Ningxia, November 2015, on the basis of the 6 influenza virus genes: A) PB2; B) PA; C) NP; D) NA; E) M; F) NS.


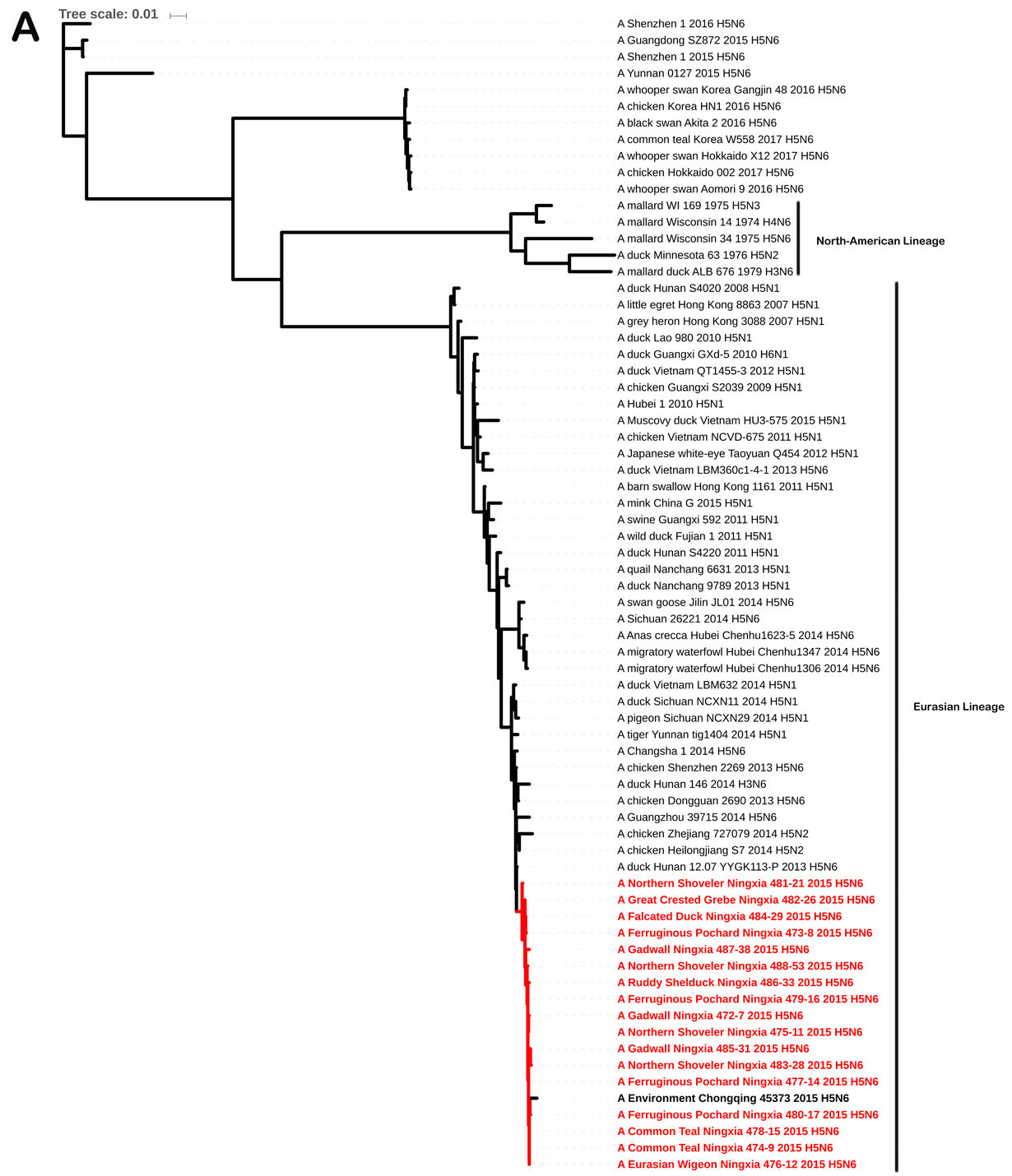


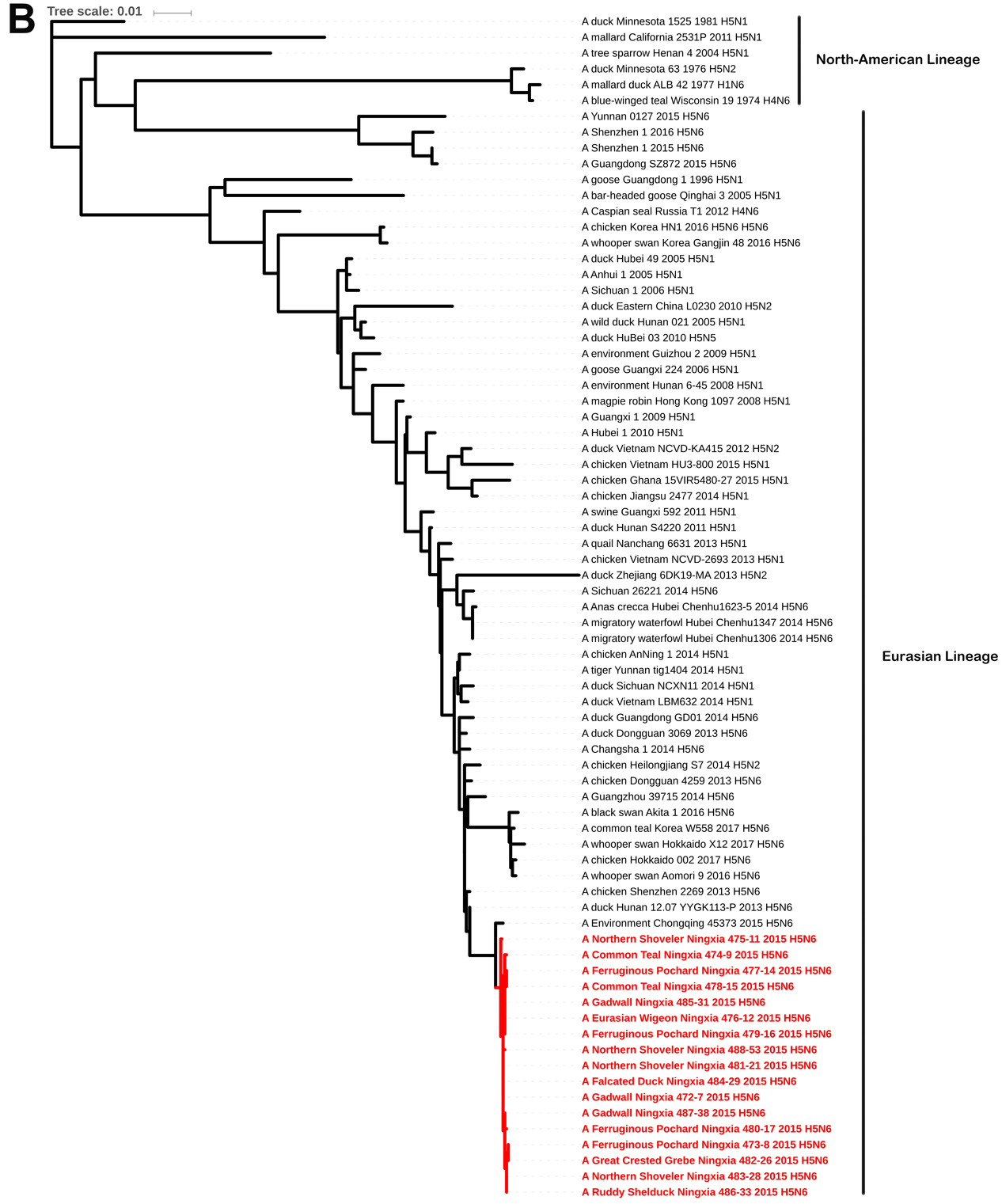


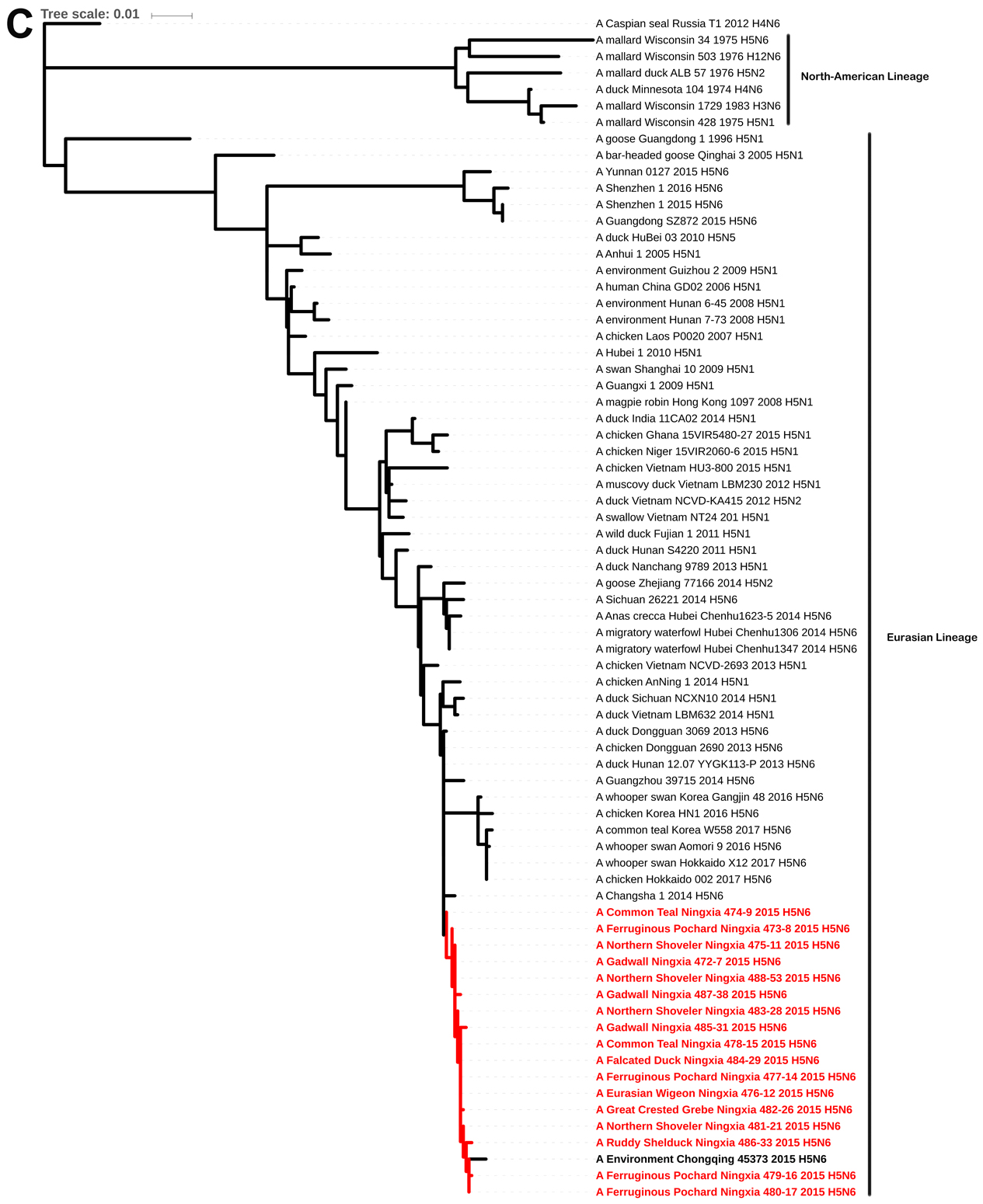


**
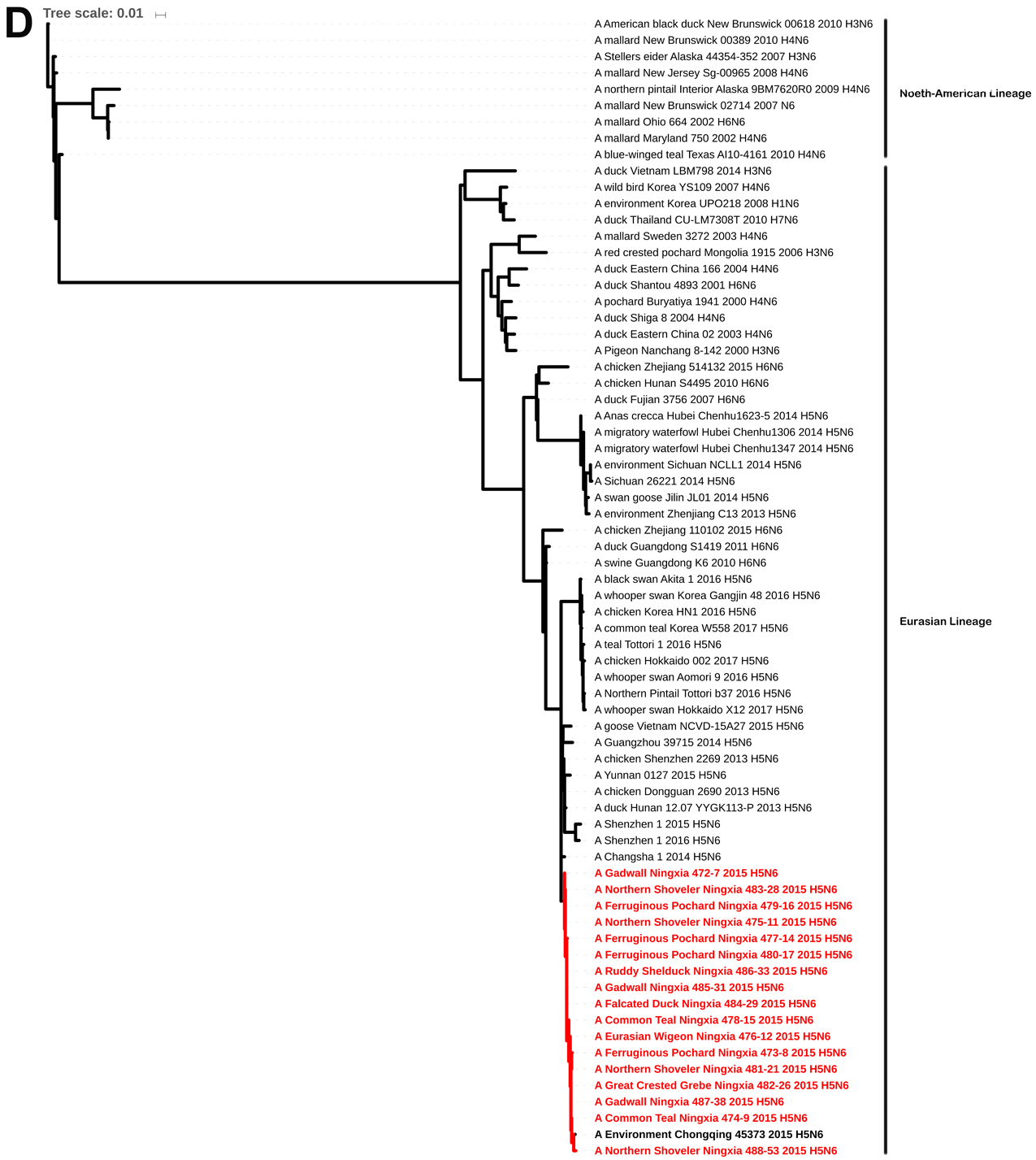
**

**
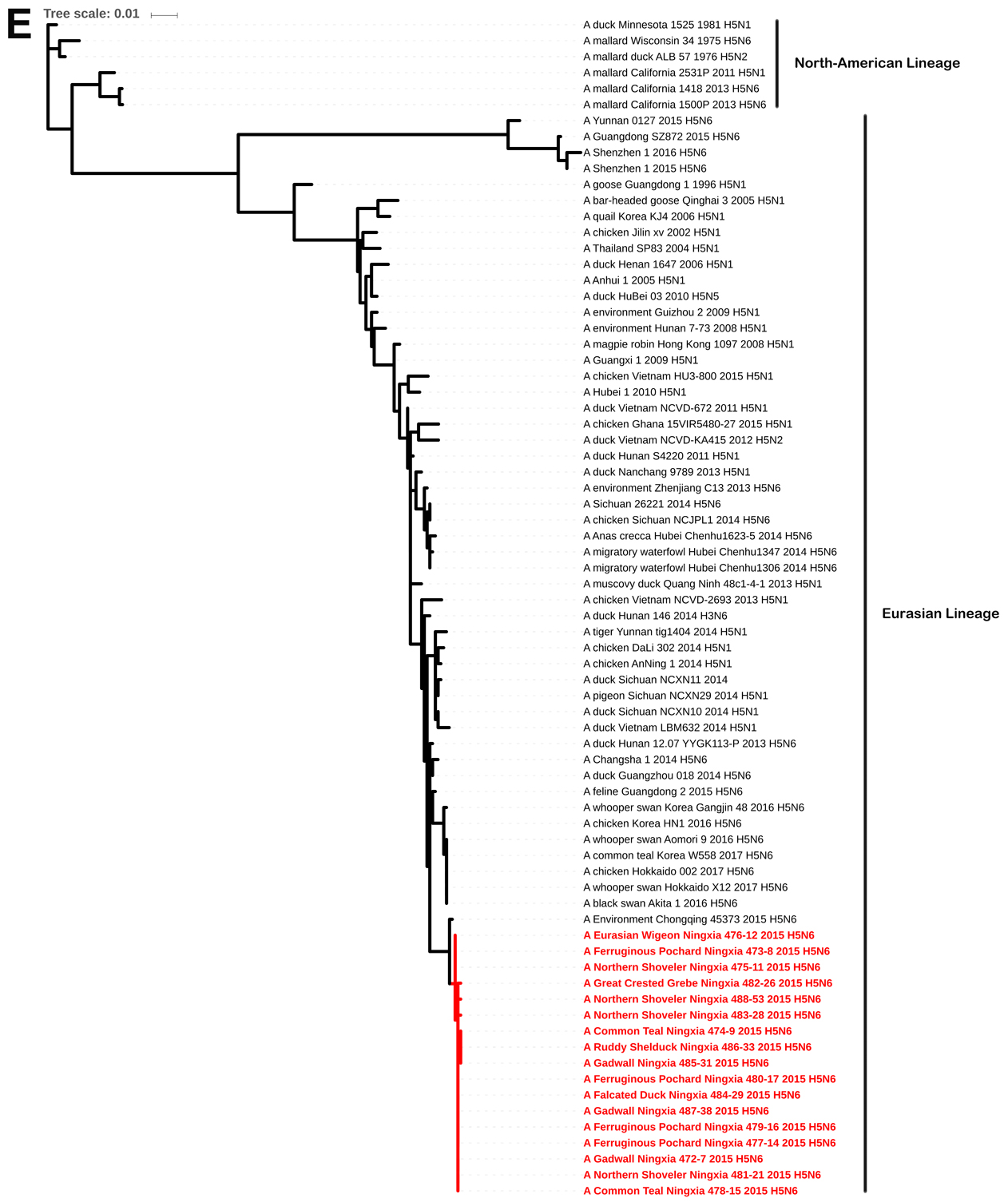
**

**
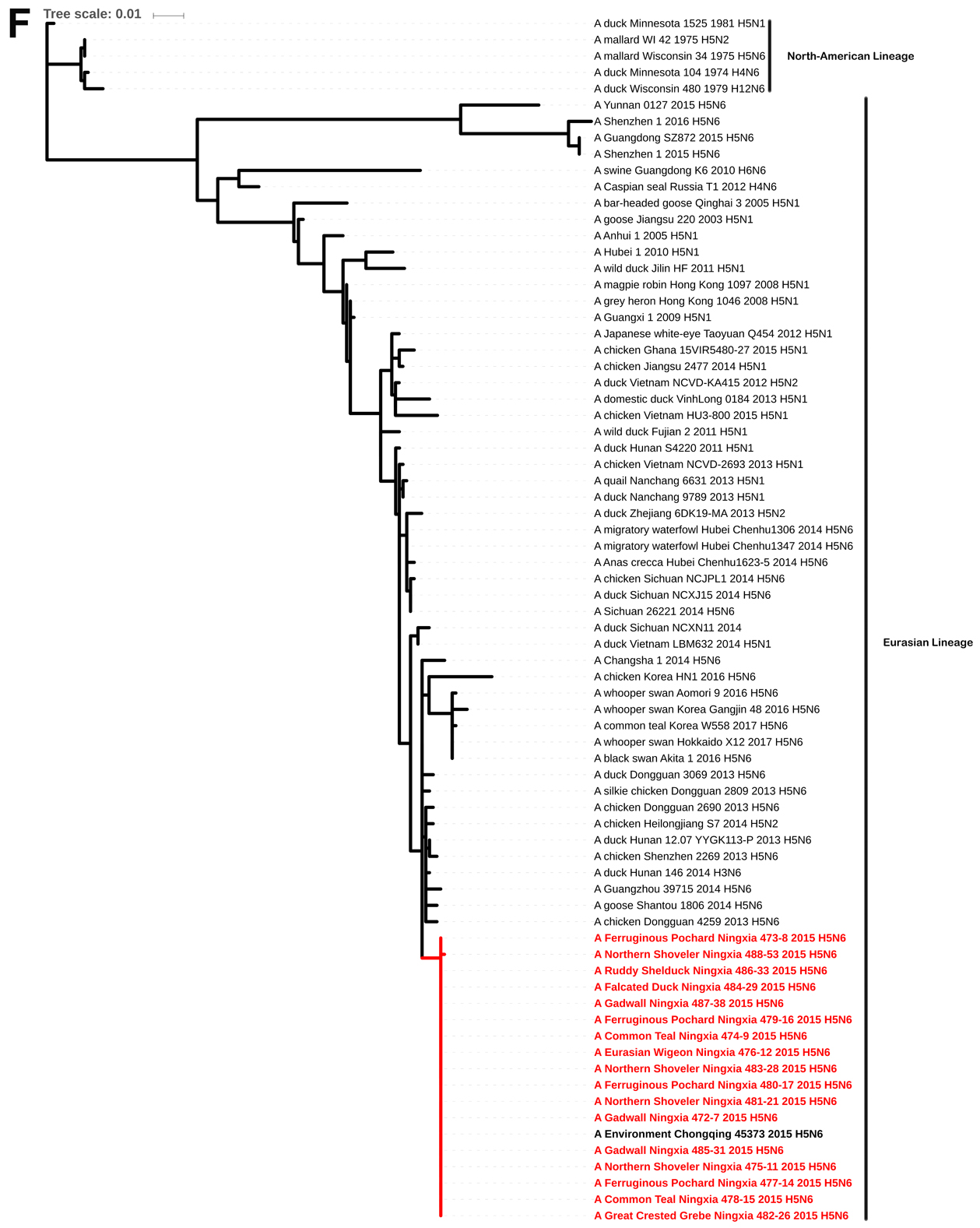
**
