# Supplementary material for: Genetics, pathogenicity and transmissibility of novel reassortant H5N6 highly pathogenic avian influenza viruses first isolated from migratory birds in western China
Source: Emerg Microbes Infect. 2018 Jan 24;7:6. doi: 10.1038/s41426-017-0001-1 (PMC5837145; doi:10.1038/s41426-017-0001-1)

**Supplementary Figure S2.** Agglutination of various red blood cell samples by the NX488-53 virus. A: Chicken red blood cells (with α-2, 3-linked sialic acid receptors and α-2, 6-linked sialic acid receptors). B: Sheep red blood cells (with only α-2, 3-linked sialic acid receptors). C: Chicken red blood cells treated with α-2, 3-sialidase (with only α-2, 6-linked sialic acid receptors). D: Chicken red blood cells treated with VCNA (no receptors).


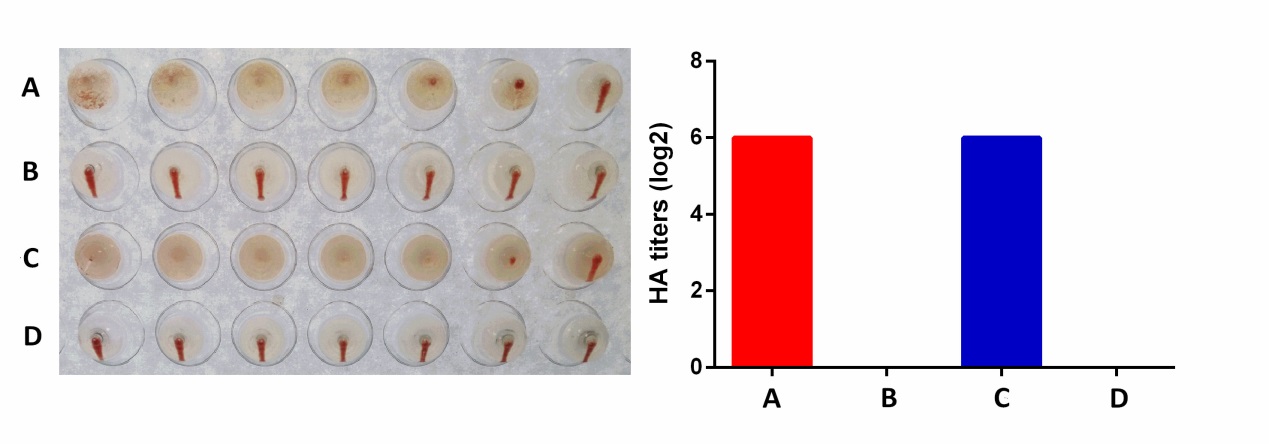

Supplement: Supplementary file 2 — Supplementary Figure S2 [file 41426_2017_1_MOESM2_ESM.docx]
