# Supplementary material for: Genetics, pathogenicity and transmissibility of novel reassortant H5N6 highly pathogenic avian influenza viruses first isolated from migratory birds in western China
Source: Emerg Microbes Infect. 2018 Jan 24;7:6. doi: 10.1038/s41426-017-0001-1 (PMC5837145; doi:10.1038/s41426-017-0001-1)

**Supplementary Figure S3.** Pathogenicity of NX488-53 virus in mice. Six mice per group were intranasally inoculated with 10^6^ EID_50_ (in 50 μL) of H5N6 virus. (A) Body weights were monitored daily for 14 days. Values represent means±standard deviation (SD) for overall body weight loss compared with initial body weight. (B) Survival percentages were calculated by observing the infected mice. (C) Mice were intranasally inoculated with 50 μL of NX488-53 virus at 10^6^ EID_50_/mL. Tissues were collected from mice (n = 3) on the indicated days post-infection (dpi), and virus titers were determined in 9-day-old specific pathogen-free embryonated eggs.
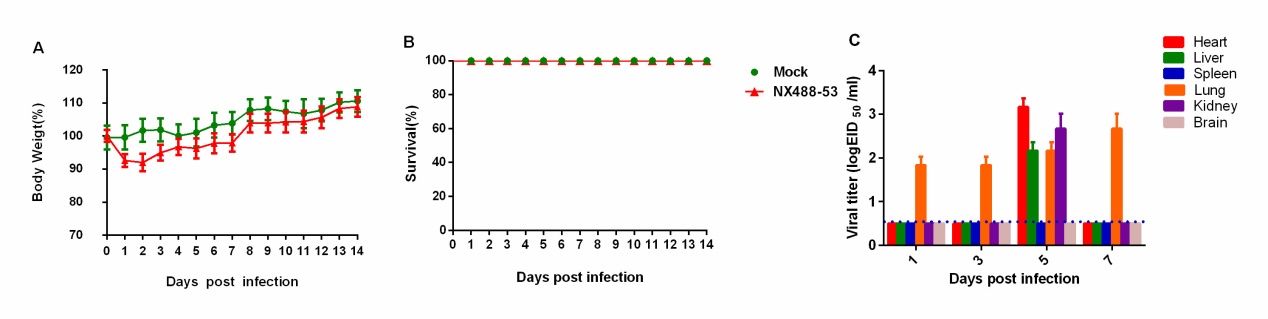

Supplement: Supplementary file 3 — Supplementary Figure S3 [file 41426_2017_1_MOESM3_ESM.docx]
