# Supplementary material for: Genetics, pathogenicity and transmissibility of novel reassortant H5N6 highly pathogenic avian influenza viruses first isolated from migratory birds in western China
Source: Emerg Microbes Infect. 2018 Jan 24;7:6. doi: 10.1038/s41426-017-0001-1 (PMC5837145; doi:10.1038/s41426-017-0001-1)

**Supplementary Figure S4.** Histopathological analysis of lungs from infected mice were fixed with formalin, embedded in paraffin and stained with hematoxylin and eosin. Images obtained using 20× magnification revealed (arrow A) a small amount of neutrophil infiltration; (arrow B) alveolar wall thickening; and (arrow C) hyperemia of alveolar wall capillaries.


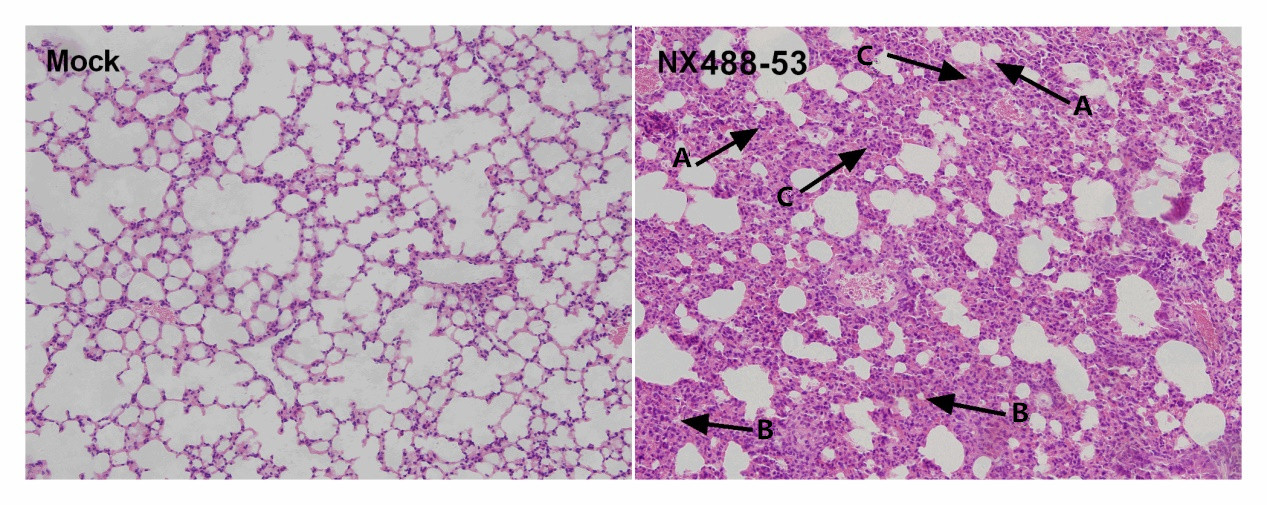

Supplement: Supplementary file 4 — Supplementary Figure S4 [file 41426_2017_1_MOESM4_ESM.docx]
