# Supplementary material for: Genetics, pathogenicity and transmissibility of novel reassortant H5N6 highly pathogenic avian influenza viruses first isolated from migratory birds in western China
Source: Emerg Microbes Infect. 2018 Jan 24;7:6. doi: 10.1038/s41426-017-0001-1 (PMC5837145; doi:10.1038/s41426-017-0001-1)
Supplement: Supplementary file 5 — Supplementary Figure S5 [file 41426_2017_1_MOESM5_ESM.docx]

**Supplementary Figure S5.** Horizontal transmission of NX488-53 viruses between guinea pigs. Groups of three guinea pigs were inoculated with 10^6^ EID_50_ (each in 200 μL) of the indicated viruses. The next day, each inoculated animal was individually paired and cohoused with a direct-contact (DC) guinea pig. An RD-contact animal was also housed in a wire-frame cage adjacent to the infected guinea pig. Nasal washes were collected every other day from all animals from day 2 of the initial infection for the detection of virus shedding. Each colored bar represents the virus titer for three guinea pigs. Dashed lines indicate the lower limit of virus detection. Error bars represent standard errors of means.
