# Supplementary material for: Genetics, pathogenicity and transmissibility of novel reassortant H5N6 highly pathogenic avian influenza viruses first isolated from migratory birds in western China
Source: Emerg Microbes Infect. 2018 Jan 24;7:6. doi: 10.1038/s41426-017-0001-1 (PMC5837145; doi:10.1038/s41426-017-0001-1)
Supplement: Supplementary file 6 — Supplementary materials [file 41426_2017_1_MOESM6_ESM.docx]

**Supplementary materials**

**Sampling**

In November 2015, 80 wild birds including 13 species were caught using a bird net during routine surveillance performed by the Monitoring Center for Terrestrial Wildlife Epidemic Diseases in Ningxia, China. Sterile swabs were used to obtain fresh and well-separated samples (1 oropharyngeal swab and 1 cloacal swab from each bird) from these 80 wild birds. Each sample was placed into a 5-mL Eppendorf tube with 2 mL of virus protection solution (phosphate-buffered saline (PBS) [pH 7.2] supplemented with penicillin, streptomycin, and 10% glycerin) and stored in viral medium at 4°C until it was transported to the laboratory; the samples were then stored at -80°C until virus isolation.

**Virus isolation, identification and genomic sequencing**

Samples were vortexed, oscillated and then centrifuged, and the collected supernatant was inoculated into 10-day-old specific pathogen-free (SPF) chicken embryos. Allantoic fluid was harvested after 72 hours of culture, and hemagglutinin (HA) activity was assayed. Viral RNA from positive samples was extracted from allantoic fluid using a QIAamp Viral RNA Mini Kit (QIAGEN, Germany), subjected to RT-PCR using the method described in the WHO manual (World Health Organization, 2002), reverse transcribed using the primer 5′-AGCRAAAGCAGG-3′ and confirmed to be from avian influenza viruses via RT-PCR^1^。

PCR amplifications were performed for subtyping of HA and neuraminidase (NA), and specific primers were utilized to amplify gene segments. PCR products for eight fragments of the H5N6 virus were sequenced using a set of specific sequencing primers. Sequence data were compiled using the SeqMan program (DNASTAR, Madison, WI, USA). RT-PCR was performed with a set of gene-specific primers. Primer sequences are available upon request.

**Data source and sequence analysis**

All reference sequences used in this study were obtained from NCBI (www.ncbi.nlm.nih.gov/genomes/FLU) and GISAID (www.gisaid.org). DNASTAR’s MegAlign was used to perform sequence homology analyses. Multiple sequence alignment was performed using Muscle. Phylogenetic analysis was performed using RAxML with 1000 bootstrap replicates, and GTRGAMMA was used as the nucleotide substitution model. Phylogenetic trees were customized and annotated by iTOL (http://itol.embl.de/index.shtml).

**Animal experiments**

**Receptor Binding Specificity Assays**

The receptor binding specificity of the NX488-53 virus was determined via HA assays involving 1% chicken red blood cells (cRBCs) and sheep red blood cells (sRBCs). The chicken red blood cells surface contains α-2, 3-linked and α-2, 6-linked sialic acid receptors. While, the sheep red blood cells surface only contains α-2,3-linked sialic acid receptors. For HA assays, α-2, 3-linked sialic acid receptors on cRBCs were enzymatically removed using an α-2, 3-specific sialidase, and only α-2, 6-linked human sialic acid receptors were retained. Other cRBCs were treated with VCNA to eliminate both α-2, 3-linked and α-2, 6-linked sialic acid receptors. Viruses were serially diluted in 50 μL PBS and mixed with 50 μL of 1% red blood cells in a 96-well plate. HA titers were measured after a 20-minute reaction at room temperature^1^.

**Mouse Challenge Studies**

Five six-week-old female BALB/c mice were obtained from Beijing Merial Vital Laboratory Animal Technology Company. To determine the morbidity and mortality, two groups BALB/c mice were anesthetized with ether and intranasal inoculated with 50μL of 10^6^ EID_50_ of the NXH5N6 virus. The weight loss and survival rate of mice in these two groups were daily monitored for two weeks. The percentage body weight change of each mouse was calculated by comparing the group average weight with their initial weight on day 0.

To detect the system spread of the NXH5N6 in mice, 12 mice were anesthetized with ether and intranasal instilled 50μL of 10^6^ EID_50_ of the virus, while another three mice intranasal instilled with PBS were as control. At 1,3,5,7 days post infection (dpi), three mice were euthanized and different tissues including lung (right lungs), heart, liver, spleen, kidney and brain were collected. The tissue samples were homogenized and centrifuged at 10000rpm. Then the supernatants were collected and inoculated into 9-day-old embryonated eggs. After 72h incubation at 37°C, the hemagglutinin activity was tested and the EID_50_ was determined by Reed Methods. The left lungs were fixed in formalin, and the fixed tissues were embedded in paraffin and were stained with hematoxylin and eosin (H & E) used for pathological examination with light microscopy.

**Guinea Pig Challenge Studies**

Guinea pigs weighing 300-350 g were obtained from Beijing Merial Vital Laboratory Animal Technology Company. For direct contact transmission studies, three guinea pigs were intranasally inoculated with 200 μL of the NX488-53 virus at 10^6^ EID_50_ and housed in a cage placed in an isolator; 24 hours later, three healthy animals were cohoused in the same cage with these three infected guinea pigs. For aerosol transmission studies, three animals were infected with 200 μL of the NX488-53 virus at 10^6^ EID_50_ and housed in three cages. After 24 hours, three healthy animals were paired with the infected guinea pigs and housed in wire-frame cages adjacent to the infected animals. The distance between the two types of cages was 5 cm. To monitor virus shedding, nasal washes were collected for all animals every other day for 7 days and assessed via virus titration in eggs.

**Ethics statements**

All animal studies were conducted in strict accordance with the guidelines for animal welfare issued by the World Organization for Animal Health. All experiments were performed in a biosafety level 2+ laboratory (an enhanced animal biosafety level 2 laboratory and a negative pressure-ventilation laboratory). Experimental protocols involving animals were approved by our institution’s animal care committee.

**References**

1 Jin H, Wang D, Sun J *et al*. Pathogenesis and phylogenetic analyses of two avian

influenza H7N1 viruses isolated from wild birds. *Front Microbiol*. 2016; 7 : 1066.

2 Sun P, Xia X, Hou X *et al*. Development of a method for identification of receptor

binding specificity of influenza virus. *Chin. J. Biol*. 2008; 21: 713-716.
