# Supplementary material for: Genetics, pathogenicity and transmissibility of novel reassortant H5N6 highly pathogenic avian influenza viruses first isolated from migratory birds in western China
Source: Emerg Microbes Infect. 2018 Jan 24;7:6. doi: 10.1038/s41426-017-0001-1 (PMC5837145; doi:10.1038/s41426-017-0001-1)
Supplement: Supplementary file 7 — Supplementary Table S1 [file 41426_2017_1_MOESM7_ESM.doc]

**Supplementary Table S1** **Molecular characterizations of H5N6 viruses.**

| **Viruses**  **(H5N6)†** | **HA (****H5 numbering)‡** | | | | | |  | **PB2** | | |  | **NA§** |  | **M1*** | | |  | **NS1*** | | | | |
| --- | --- | --- | --- | --- | --- | --- | --- | --- | --- | --- | --- | --- | --- | --- | --- | --- | --- | --- | --- | --- | --- | --- |
|  | Cleavage sites | 94 | 123 | 133 | 224 | 226 |  | 89* | 627^＆^ | 701^＆^ |  | NA del,  59-69 |  | 30 | 139 | 215 |  | 80-84  del | 42 | 87 | 98 | 101 |
| NX488-53 | RERRRKR/GLF | N | P | A | Q | G |  | V | E | D |  | YES |  | D | T | A |  | YES | S | E | F | M |
| CQ45373 | RERRRKR/GLF | N | P | A | Q | G |  | V | E | N |  | YES |  | D | T | A |  | YES | S | E | F | M |
| CS1 | RERRRKR/GLF | N | P | A | Q | G |  | V | E | D |  | YES |  | D | T | A |  | YES | S | E | F | M |

†NX488-53:A/Northern Shoveler/Ningxia/488-53/2015(H5N6)；CQ45373: A/Environment/Chongqing/45373/2015 (H5N6); CS1: A/Changsha/1/2014(H5N6).

‡D94N, S123P, S133A, Q224L and G226S mutations in HA have been associated with increased binding to human-like receptor (α-2, 6-sialic acid).

^＆^E627K and D701N mutations have been associated with improved replication of avian influenza virus in mammals.

§NA stalk deletion has been associated with enhanced pathogenicity in mice.

*L89V, N30D, T139A, T215A, 80-84 deletion, P42S, D87E, L98F and I101M have been associated with increased virulence in mice.
